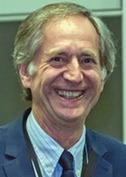# New Designs for Phototherapeutic Transition Metal Complexes

**DOI:** 10.1002/anie.201905171

**Published:** 2019-09-24

**Authors:** Cinzia Imberti, Pingyu Zhang, Huaiyi Huang, Peter J. Sadler

**Affiliations:** ^1^ College of Chemistry and Environmental Engineering Shenzhen University Shenzhen 518060 China; ^2^ School of Pharmaceutical Science (Shenzhen) Sun Yat-sen University Guangzhou 510275 China; ^3^ Department of Chemistry University of Warwick Coventry CV4 7AL UK

**Keywords:** anticancer, metal complexes, photoactivated chemotherapy, photodynamic therapy

## Abstract

In this Minireview, we highlight recent advances in the design of transition metal complexes for photodynamic therapy (PDT) and photoactivated chemotherapy (PACT), and discuss the challenges and opportunities for the translation of such agents into clinical use. New designs for light‐activated transition metal complexes offer photoactivatable prodrugs with novel targeted mechanisms of action. Light irradiation can provide spatial and temporal control of drug activation, increasing selectivity and reducing side‐effects. The photophysical and photochemical properties of transition metal complexes can be controlled by the appropriate choice of the metal, its oxidation state, the number and types of ligands, and the coordination geometry.

## Graphical abstract

**Light irradiation** can provide spatial and temporal control of drug activation, increasing selectivity and reducing side‐effects. This Minireview summarizes new designs for light‐activated transition metal complexes which offer photoactivatable prodrugs with novel targeted mechanisms of action for use in photodynamic therapy (PDT) and photoactivated chemotherapy (PACT).

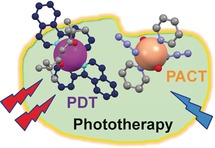

## 1 Introduction

Nature uses light to promote and control some of the most crucial reactions, from photosynthesis, converting light and carbon dioxide to oxygen and sugar, to the synthesis of vitamin D in our skin. In the same way, the power of light can be harnessed for medical applications. The use of light in medicine (phototherapy) dates back to ancient Egypt, India and China for the treatment of skin diseases. In the 19^th^ century, Niels Ryberg Finsen used light for the treatment of smallpox and lupus vulgaris, which earned him the 1903 Nobel Prize in Physiology.^1^ Later in the 20^th^ century, light was used to stimulate tissue healing (photobiostimulation), opening a new avenue in phototherapy, by deliberately triggering intracellular photobiochemical reactions following light absorption by endogenous chromophores such as porphyrins.^2^

In contrast, the use of light in combination with exogenous chemicals, to control the therapeutic activity of agents selectively in space and time, has been explored only in recent decades. Photofrin, based on oligomers of porphyrin units, was the first approved photosensitizer for clinical photodynamic therapy, in 1993.^3^ Importantly, the potential for activating a drug selectively in the tissue of interest, thus minimising side effects, is the obvious, but not unique advantage of this strategy. Because photoactivatable molecules exert their therapeutic action via excited states, the mechanism of action is often significantly different from traditional chemotherapeutics and less subject to cross‐resistance with existing pharmaceuticals.^4^ This potential of photoactivatable therapeutics, combined with the development of laser‐ and LED‐based technologies for effective delivery of light to different tissues, has led to the development and clinical investigation of several photodynamic therapy agents, including four organic tetrapyrrolic derivatives (Photofrin, Chlorin e6, Visudyne and Foscan), and four metal‐based photosensitizers: WST11 (Pd^II,^, approved for vascular‐targeted of prostate cancer),^5^ Lutex (Lu^III^, lutetium texaphyrin, for cervical intraepithelial neoplasia), Purlytin (Sn^IV^, age‐related macular degeneration), and TLD1433 (Ru^II^, non‐muscle invasive bladder cancer).^5^, ^6^, ^8^

Although the application of metal‐based complexes for phototherapy is at a relatively early stage compared to organic photosensitizers, the chemical know‐how accumulated over years of experience with cisplatin and other metal‐based drugs can accelerate development of photoactivatable complexes. In turn, progress in this area provides new scope for the application of bioinorganic chemistry in medicine. Here we review recent advances in the development of metal‐based photoactivatable agents for both photodynamic therapy (PDT) and photoactivated chemotherapy (PACT), recognising opportunities and challenges for the clinical translation of these agents. A few agents from our laboratories will be discussed in detail as examples of promising candidates for PDT and PACT. More general comprehensive reviews are available elsewhere.^6^

## 2 PDT Agents

Photodynamic therapy requires the simultaneous presence of three essential components: 1) a photosensitizer, 2) oxygen, and 3) light. Absorption of light excites the photosensitizer from the ground electronic state to an excited singlet state, which can convert to a long‐lived triplet excited state by intersystem crossing (ISC). Quenching of this state by biomolecules (type I reaction) or directly by molecular oxygen ^3^O_2_ (type II reactions) produces oxygen radicals and singlet oxygen (^1^O_2_), respectively. Both these reactive oxygen species (ROS) can induce severe oxidative damage to cellular biological molecules, resulting in vascular injury and tumor cell death. Owing to the highly reactive nature of singlet oxygen (^1^Δ_g_ state 95 kJ mol^−1^ higher in energy than ^3^O_2_), type‐II processes are generally considered as the main photosensitization mechanism in PDT.^7^ Importantly, the photosensitizer returns to the ground state upon quenching and becomes newly available for another cycle. Therefore, in principle, only a catalytic amount of photosensitizer is required to achieve therapeutic efficacy.

**Figure 1:**
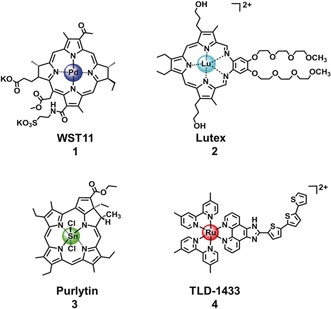
Metal‐based photosensitizers in clinical trials for PDT.

The design of photosensitizers has improved significantly in recent years through enhancement of their photophysical and biological properties. Figure 1 shows some of the most promising metal‐based photosensitizers recently developed for clinical use, including tetrapyrrolic derivatives of Pd^II^ (WST11, **1**), Lu^III^ (Lutex, **2**), and Sn^IV^ (Purlytin, **3**).^5^, ^6^ Notably, the presence of a Pd centre in WST11 improves stability and excited state reactivity and, at the same time, greatly enhances the ISC rate of the photosensitizer.^5^ Another advantage of introducing a non‐essential metal is that it can allow quantification and localization of the photosensitizer in cells and tissues by a variety of techniques, including X‐ray absorption spectroscopy (XAS) and inductively coupled plasma‐mass spectrometry (ICP‐MS). Furthermore, if the metal complexes are luminescent, their cellular localization can often be precisely determined by confocal microscopy.^8^

Ruthenium(II) polypyridyl complexes, including TLD1433 (**4**; Figure 1), the first metal‐based photosensitizer without a tetrapyrrolic moiety to enter clinical trials for non‐muscle invasive bladder cancer PDT (ClinicalTrials.gov, identifier NCT03053635),^6^ are attracting significant attention as PDT photosensitizers, as are luminescent polypyridyl complexes of Ir^III^, Os^II^ and Re^I^.^9^

In the next sections, we present examples of promising new photosensitizers and consider in more detail their mechanism of action, with particular focus on their interaction with proteins upon irradiation. We also highlight efforts to improve delivery of metal‐based photosensitizers to cancer cells by attaching them to serum proteins, and how this can result in targeted delivery and their localization in specific subcellular organelles.

### 2.1 Protein Oxidation by Metal‐based Photosensitizers

Owing to their abundant and ubiquitous presence in cells and to their reactivity towards ^1^O_2_ and other ROS, proteins are generally regarded as the main target of photodynamic therapy, followed by lipids and nucleic acids.^10^ In particular, cysteine, methionine, histidine, tyrosine and tryptophan amino acid residues are especially susceptible to oxidative damage by ^1^O_2_.^11^ Although investigations of the effects of PDT agents on proteins have mainly revolved around organic photosensitizers, some reports also discuss interactions with metal‐based PDT agents.

**Figure 2:**
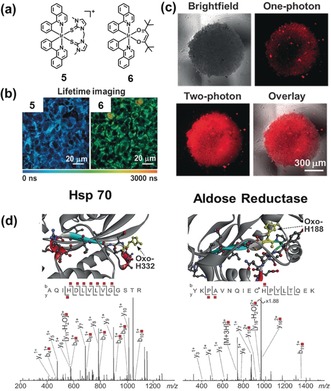
Ir^III^ complexes for one‐ and two‐photon PDT. a) Chemical structures of **5** and **6**. b) Phosphorescence lifetime imaging of living A549 lung cancer cells treated with **5** and **6**, respectively. c) One‐ and two‐photon phosphorescence of A549 spheroids treated with **6**. d) Structures and LC‐MS/MS analysis of the oxidized peptides of Hsp 70 (PDB ID: 3ATV) and aldose reductase (PDB ID: 1US0). Images reproduced with permission from Ref. 12. Copyright 2017, John Wiley & Sons.

The strongly luminescent octahedral Ir^III^ complexes **5** and **6** (Figure 2) exhibit high PDT efficiency via both 1‐photon and 2‐photon light activation, with their extremely long phosphorescence lifetimes in cancer cells giving rise to a high ^1^O_2_ quantum yield.^12^ Notably, the O^O chelated complex **6** showed low dark toxicity to cells, but became potently cytotoxic upon irradiation with very low doses of visible light. Moreover, complex **6** exhibited sub‐micromolar IC_50_ values towards 3D multi‐cellular tumor spheroids (models for solid tumors) when irradiated with 750 nm two‐photon near infrared light. Their effects on proteins in A549 lung cancer cells, was investigated using liquid chromatography‐tandem mass spectrometry, which revealed specific oxidation of histidine residues to 2‐oxo‐His in the key proteins aldose reductase and heat‐shock protein‐70 (Hsp‐70) after photoactivation of complex **6**. Notably, the oxidative stress induced during the photoactivation increased the levels of enzymes involved in the glycolytic pathway. This might arise from a switch in energy (ATP) production from oxidative phosphorylation (reduction of O_2_ to H_2_O in mitochondria) to glycolysis (conversion of glucose to pyruvate in the cytoplasm). The results suggest that iridium complexes might target specific proteins in cancer cells and induce oxidation irreversibly upon photoactivation, contributing to efficient PDT.

**Figure 3:**
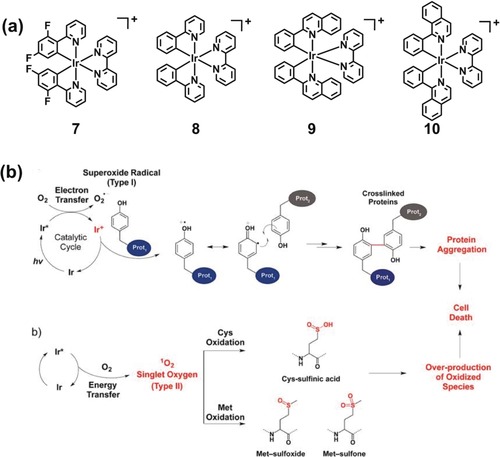
Protein modification pathways induced by Ir^III^ complexes in cancer cells. a) Chemical structure of Ir^III^ photosensitizers **7**–**10**. b) Cross‐linking and oxidative damage pathways for protein modification observed upon photoactivation of Ir^III^ complexes. Image reproduced with permission from Ref. 13. Copyright 2016 American Chemical Society.

Kwon et al. have recently reported structure–activity relationships for related cyclometalated Ir^III^ complexes **7**–**10** (Figure 3), for one‐ and two‐photon PDT and targeting of the endoplasmic reticulum (ER).^13^ This organelle plays an important role in protein synthesis, lipid manufacture and metabolism, production of steroid hormones, and detoxification. ER damage leads to ER stress response, which can cause cell death via apoptosis involving the mitochondrial pathway. Importantly, the favorable triplet state energy of Ir^III^ complexes and high emission quantum yield of complex **9** resulted in a remarkably high singlet oxygen quantum yield in water (95 %), along with superoxide production. Confocal microscopy revealed that **9** predominantly localized in the ER, and exhibited a long luminescence lifetime (500 ns) in cancer cells. Upon low‐dose “sunlight” irradiation (1.0 J cm^−2^), **9** showed photocytotoxicity toward SK‐OV‐3 ovarian and MCF‐7 breast cancer cells. The potential use of **9** as a two‐photon PDT agent was further investigated by evaluating the morphological changes of SK‐OV‐3 cells, upon irradiation with an 860 nm laser. Notably, cell shrinkage was observed as a function of time also in these conditions, demonstrating two‐photon‐induced phototoxicity of **9**. The specific oxidative reactions arising from attack on endogenous proteins are attributed to both singlet oxygen (oxidation of methionine residues) and superoxide radicals (photo‐induced cross‐linking of metabolic proteins). Overall, ROS generation resulted in protein dysfunction and triggered cancer cell death.

### 2.2 Protein‐assisted Delivery of Metal‐based Photosensitizers

The natural association of metal‐based drugs with human serum proteins whose transmembrane transporters are upregulated in malignant tissues, is a well‐known strategy to achieve selective accumulation of metal coordination complexes in tumors rather than in healthy tissues. Accordingly, deliberate attachment of metal‐based photosensitizers to serum proteins, either through covalent linking or non‐covalent interaction, has been recently investigated as a delivery strategy for PDT agents to target cancerous tissues effectively.

**Figure 4:**
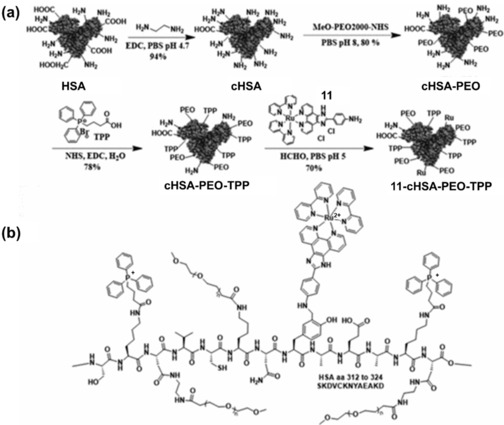
HSA‐bound Ru^II^ complex (**11**–cHSA–PEO–TPP) for mitochondrial targeting. a) Synthetic route for **11**–cHSA–PEO–TPP. b) Schematic illustration showing part of the HSA sequence with the PEO, TPP groups attached to, e.g., lysine, and **11** conjugated to tyrosine residues. Image adapted with permission from Ref. 15. Copyright 2017 American Chemical Society.

Ruthenium(II) photosensitizers are known to associate with albumin and transferrin in serum, enabling highly efficient receptor‐mediated transport into cancer cells.^14^ Lilge and colleagues have recently developed rutherrin by combining TLD1433 and transferrin, which not only enhances effective cancer targeting, but also improves photosensitizer efficiency, with a significantly increased absorbance in the green‐to‐near infrared (NIR) range.^14^ Weil and colleagues have reported **11**‐cHSA‐PEO‐TPP, (Figure 4) as a delivery system for the ruthenium‐based photosensitizer **11**, based on the blood protein serum albumin (HSA, the most abundant blood serum protein, ca. 0.6 mm) made polycationic and decorated with multiple triphenylphosphonium (TPP) groups to target mitochondria, and polyethylene oxide (PEO) chains to improve water solubility.^15^ In addition to mitochondrial localization, the **11**–cHSA–PEO–TPP conjugate exhibited significantly improved phosphorescence intensity and lifetime, as well as enhanced ^1^O_2_ quantum yields compared to the parent Ru^II^ complex (**11**), using blue (470 nm) light.

**Figure 5:**
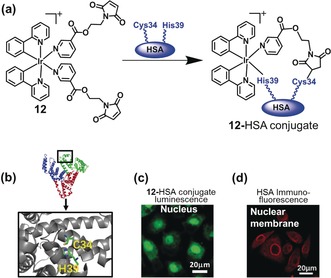
Organoiridium–human serum albumin photosensitizer (**12**–HSA conjugate) for PDT. a) Conjugation of **12** to HSA. b) Structure of HSA (PDB:5IJF); Cys 34 and His 39 are labelled. c) Confocal microscopy images of living A549 cells incubated with **12‐**HSA conjugate. d) Immunofluorescence staining of HSA in cells exposed to **12‐**HSA conjugate. Images reproduced with permission from Ref. 16.

To improve the cancer targeting ability and photochemical properties of our organoiridium photosensitizer **12** (Figure 5), we anchored it to the free thiol of HSA at cysteine‐34 using maleimide‐functionalized ligands. The conjugate exhibited significant enhancement of phosphorescence intensity compared to the complex alone, together with surprising nuclear localization of the Ir^III^ complex.^16^ Titration studies indicated that the iridium(III) complex interacts with histidine, resulting in an emission enhancement of about 37‐fold. In contrast, no significant luminescence enhancement was observed on interaction with other amino acids, including Cys. This suggested that, after binding of Ir to Cys 34, one sterically‐hindered monodentate maleimide ligand is released, being displaced by a histidine side‐chain, likely nearby His 39. Ir^III^ complexes with weakly bound ligands are known to bind strongly to amino acids/proteins through ligand substitution reactions, especially to histidine and histidine‐rich proteins such as HSA.^17^

The **12**–HSA conjugate exhibited a longer phosphorescence lifetime than complex **12** and remarkably higher ^1^O_2_ generation quantum yield. Notably, the **12**–HSA conjugate showed little toxicity in the dark, but potent photocytotoxicity with significant selectivity for cancer cells, including cancer cell spheroids, over healthy cells. Surprisingly, upon uptake into the cells, HSA appears to facilitate delivery of the Ir into the nucleus. However, HSA itself cannot penetrate the nuclear membrane, and Ir is probably released from the **12**–HSA conjugate and then migrates into the nucleus. In contrast to more traditional Ir‐based conjugates, **12**–HSA conjugate seems to be the first example of an HSA‐functionalized iridium conjugate which targets cell nuclei.

A supramolecular approach to protein‐assisted delivery of PDT agents has also recently been explored. Liu and co‐workers synthesised a poly‐phenanthroline Ru^II^ photosensitizer with 3 pendant cyclodextrin groups which strongly associate with adamantane‐functionalized transferrin molecules via non‐covalent binding of the adamantane group to the cyclodextrin cavity.^18^ Notably, the resulting supramolecular assembly exhibited selective accumulation in A549 lung cancer cells through transferrin receptor‐mediated endocytosis, displaying promising PDT efficiency. Interestingly, glutathione was found to have a protective role in A549 cells during irradiation treatment with this supramolecular system.

## 3 Photoactivated Chemotherapy (PACT) Agents

Effective PDT relies on the presence of oxygen in the target tissue. However, tumor masses often contain hypoxic regions where, owing to the suboptimal vascularization, the concentration of oxygen is remarkably low.^19^ Photoactivated chemotherapy has the potential to overcome this limitation.^20^ In PACT, light activation of otherwise inert metal complexes, can produce cytotoxic species in a controlled manner. Four main classes of PACT agents can be identified based on their mechanism of action, although the chemical complexity of these compounds can rarely be contained by such clear‐cut boundaries, as recently shown for some Ru^II^ complexes.^21^

1) **Photoinduced electron transfer**: Complexes with inert low‐spin d^6^ metal centres such as Pt^IV[22]^ and Co^III[23]^ can be activated by irradiation into their ligand‐to‐metal charge‐transfer (LMCT) bands causing the permanent transfer of electrons from the ligand to the metal centre, and ligand dissociation.^4^ The therapeutic effect can be ascribed to the reduced metal complex, the released ligands, or both. A different type of photoinduced electron transfer is observed for polyazaaromatic Ru^II^ complexes, where light irradiation oxidizes the metal centre generating an excited‐state highly‐oxidising Ru^III^ species that can interact with DNA or other biomolecules.^24^

2) **Photosubstitution**: Light irradiation of inert d^6^ Ru^II^,^25^ Rh^III[26]^ and Ir^III[27]^ complexes can create an excited metal‐to‐ligand charge‐transfer triplet state (^3^MLCT), which rapidly interconverts to a highly dissociative excited metal‐centred triplet state (^3^MC). This results in the release of a ligand and generation of reactive species, which can both contribute to the therapeutic effect.^21^ Complexes with different metals/configuration, including a Pt^II^‐curcumin derivative, have also been investigated for photosubstitution reactions.^28^

3) **Bioactive ligand release**: Several complexes based on Mn^I^, Re^I^ and Cr^III^ centres can release, upon irradiation, small bioactive molecules such as NO^29^ and CO.^30^ Ru^II^ complexes acting as photocages for enzyme inhibitors^31^ or neurotransmitters^32^ have also been investigated.

4) **Ligand photocleavage**: For this class of complexes, photoactivation is ligand‐centred and results in the formation (ligand photoswitch) or release (ligand photocleavage) of a cytotoxic species. Examples of ligand photocleavage include Pt^II^, Ru^II^, Re^I^ and Ir^III^ complexes, while ligand photoswitch has been reported for Pt^II^ agents.^33^

### 3.1 Azido Pt^IV^ Agents

The photochemistry and photobiology of azido Pt^IV^ PACT complexes have been studied in detail in our laboratory. In general, octahedral Pt^IV^ complexes are inert low‐spin 5d^6^ prodrugs for Pt^II^ anticancer agents, being reduced by intracellular reduction (e.g. by ascorbate or thiols such as glutathione).^34^ However, extracellular reduction (e.g. in blood) can limit their therapeutic efficacy and increase side effects. For example, the Pt^IV^ prodrug iproplatin has more severe side effects than carboplatin with no enhanced chemotherapeutic effects.^35^ Accordingly, given also the promising clinical achievement of PDT in light‐controlled oncotherapy, there is interest in controlling the reduction and activation of Pt^IV^ prodrugs by light irradiation.

**Figure 6:**
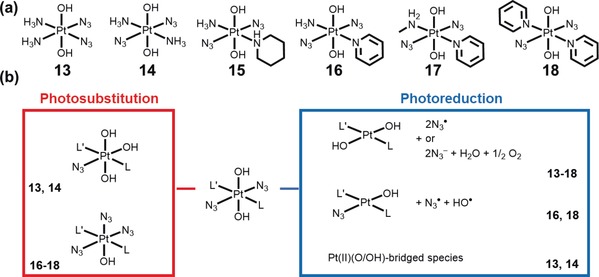
a) Chemical structures of selected azido Pt^IV^ complexes, and b) various photoproducts detected upon irradiation (the complexes for which a particular photoproduct has been detected experimentally are indicated). Charges are omitted for clarity.

The photoreduction of azido Pt^IV^ complexes was first explored by Vogler who observed light‐mediated reduction of *trans*‐[Pt(N_3_)_2_(CN)_4_]^2−^ with release of azidyl radicals.^36^ Our group has focused on the design of complexes with the general formula [Pt(N_3_)_2_(OH)_2_(L)(L′)] where L and L′ are a(m)mine ligands which can be *cis* or *trans*, with *trans* hydroxide ligands, arbitrarily defined as axial ligands. A few of the most studied complexes are shown in Figure 6 a. Unexpectedly, cell viability studies revealed an increased photocytotoxicity of the *trans* diammine complex **14** compared to the *cis* isomer **13**.22a Related *trans* complexes were also more active,^37^ suggesting that this class of complexes has different modes of action compared to conventional *cis*‐diam(m)ine Pt^II^ drugs. The potency of *trans* Pt^IV^ diazido agents increases for complexes bearing a bulky amine substituent, as in **15** 38 or **16**.22b Notably, an in vivo experiment with complex **16** demonstrated the efficacy of this complex in slowing the growth of OE19 oesophageal cancer xenografts in nude mice using low doses of **16** and short irradiation times with blue light.^39^ Complex **18** with two pyridine ligands exhibited the highest cytotoxicity upon irradiation.22d

A relatively straightforward pathway was initially proposed for the photoreduction of Pt^IV^ azido complexes via reductive elimination, to release two azidyl radicals (which could form 3 molecules of N_2_) and yield a square‐planar Pt^II^ species. Although this mechanism was supported by UV‐vis measurements showing a rapid decrease in intensity of the band assigned to the Pt←N_3_ LMCT upon irradiation, different analytical techniques revealed that several decomposition pathways are available depending on the structure of the complex and on the solution environment. The photo‐activated processes are not limited to photoreduction, but also include photosubstitution and, for *cis* complexes, photoisomerisation.^40^ Pathways for a typical *trans* photoactivatable Pt^IV^ complex are summarised in Figure 6 b. Notably, all the observed photoproducts have lost at least one azide ligand. Liberation of the ammine ligand was also observed for L=NH_3_, but not for bulkier secondary or tertiary amines such as piperidine and pyridine, respectively.^38^, ^41^ The number of possible photoreduction pathways seemed somewhat reduced for complexes with more sterically‐hindered amines, with three photoproducts only observed for **16,** 42 and one for **15**.^43^

The efficiency of photodecomposition processes for these diazido Pt^IV^ complexes depends intimately on the types of ligands and their stereochemistry. Complexes with at least one bulky amine are reduced more rapidly,^38^ and, notably, *trans* complexes can be reduced at longer wavelengths and to a greater extent than their *cis* analogues.22a This was rationalised by DFT calculations, showing how low‐lying excited states have a lower energy for *trans* complexes.^44^

Investigating interactions of biomolecules with these complexes upon irradiation is key to understanding the photoproducts that could form inside cells. Unexpectedly, irrespective of the azido Pt^IV^ complex, guanosine monophosphate (GMP) markedly accelerated the photoreduction process, likely by trapping the resulting Pt^II^ species.^43^ Similar to formation of bis(GMP) adducts by cisplatin, [Pt(L)(L′)(GMP‐N7)_2_]^2+^ readily formed, and more rapidly for the *trans* than the *cis* isomers.22a, 45 However, other adducts were also formed including the mono adduct [Pt(N_3_)(L)(L′)(GMP‐N7)]^+^, for **16**–**18** at short irradiation times.22b, 22d Notably, **17** induced oxidation of guanine upon irradiation, likely via singlet oxygen generation even under an argon atmosphere and, remarkably, formation of the ammine complex [Pt(NH_3_)(py)(MA)(8‐OH‐GMP)] probably via a nitrene intermediate.^46^

DNA platination is observed upon irradiation, but not in the dark, and is slower for complexes carrying bulky ligands.^38^, ^42^ Bulky *trans* complexes generate fewer plasmid DNA intrastrand crosslinks than cisplatin, but cause much larger DNA unwinding.22b, 38, 47 Also they form adducts that are less effectively removed by DNA repair mechanisms,22b and stall RNA pol II transcription to a greater extent than cisplatin.^47^

The photoreduction of complex **13** in the presence of dimethyl sulphide and 1‐methylimidazole (models for methionine and histidine side chains, respectively), suggested that these complexes might also attack proteins upon irradiation.^40^, ^41^ Furthermore, sulfur oxidation and evolution of O_2_ suggested that ROS may be produced in the process.41a High resolution mass spectrometry studies with complex **18** confirm its ability to platinate peptides (subP and bombesin) and proteins (thioredoxin) upon irradiation, and to oxidise some amino acid residues (Met, Trp and Cys).^48^ Azidyl radicals liberated by photoreduction of **18** can extract an electron from tryptophan to generate Trp radicals which can be trapped and detected by EPR.^49^ This suggests that electron transport pathways in cells might be attacked, although the quenching of azidyl radicals by Trp itself can have a protective effect against phototoxicity in cells. A similar effect was observed for the antioxidant melatonin.49a

### 3.2 Mechanisms of Cytotoxicity for Azido Pt^IV^ Complexes

Although initially conceived as prodrugs for cisplatin and related conventional anticancer drugs, diazido Pt^IV^ complexes possess unique mechanisms of action; the *trans* complexes have higher potency compared to their *cis* isomers, and lack cross‐resistance with cisplatin. Different mechanisms of cytotoxicity are involved especially for *trans* diazido complexes.^22^ In particular for complex **16**, *trans*‐[Pt(N_3_)_2_(OH)_2_(NH_3_)(pyridine)], significant DNA cross‐linking is observed only at a concentration of 2× IC_50_.22b

Importantly, the lipophilicity of these diazido complexes does not correlate with cellular accumulation of platinum (in the dark), and this, in turn, is not correlated with the photocytotoxicity.^50^ On the other hand, Pt accumulation in cells upon irradiation appears to correlate with cytotoxicity for complexes **13**, and **16**–**18**.^38^, ^47^, ^51^ Hence photoexcited state structure–activity relationships and the nature of the photoproducts are probably key to understanding biological potency.

In addition to platinum‐mediated toxicity, nitrogen/oxygen radical species generated upon irradiation can also contribute to the cytotoxicity of these complexes. Inhibition of thioredoxin following treatment with **18**, *trans*‐[Pt(N_3_)_2_(OH)_2_(pyridine)_2_], and irradiation, has been attributed to oxidation rather than platination suggesting an important role for ROS in the toxicity.48b Furthermore, a significant production of RNS/ROS is observed for 6 h after irradiation of cancer cells treated with **16** or **18**.^43^, 48b Delivery of azide ions to cancer cells might also play a role in the biological activity of these complexes, given the ability of azide to inhibit a wide range of heme proteins, but this has been little explored so far.^52^

Intriguingly, upon irradiation, **13** *trans,trans,cis*‐[Pt(N_3_)_2_(OH)_2_(NH_3_)_2_] dramatically altered the morphology of cancer cells causing ballooning followed by cellular shrinkage, loss of contact with neighbouring cells, and detachment from the cell culture flask. Although nuclear packing was observed, the absence of other hallmarks of apoptosis (such as blebbing and cellular fragmentation) suggested a different mechanism of cell death for this complex.^51^ Similarly, compound **16** did not show apoptotic markers such as caspase 3 and 7 activation, or redistribution of phosphatidyl serine. Also, in contrast to cisplatin, no arrest of the cell cycle was detected for this compound. Rather, **16** seemed to induce autophagic cell death, showing increased levels of proteins associated with this self‐eating process.^39^

Involvement of the immune system in the mechanism of action of several metal anticancer complexes has been recently recognised.^53^ Notably, oxaliplatin, but not cisplatin, promotes immunogenic cell death in vivo,^54^ likely due to its different mechanism of action, relying on generation of ribosome biogenesis stress rather than DNA damage.^55^ The role of immunogenic cell death in the mechanism of action of photoactivatable Pt^IV^ complexes is currently being explored.

### 3.3 Improving Delivery of Azido Pt^IV^ Complexes

The two hydroxido ligands of complexes **13**–**18** allow for their facile derivatization via ester bond formation. In particular, the succinate derivative obtained by reaction of those complexes with succinic anhydride is a very useful intermediate to link such complexes to targeting moieties or delivery platforms in order to improve their accumulation in target tissues.

**Figure 7:**
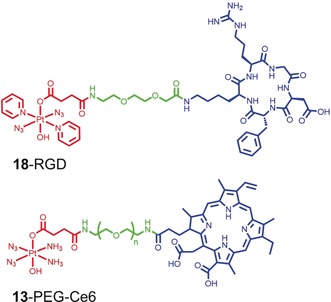
Examples of Pt^IV^ azido agents derivatized in the axial position via a succinate intermediate. The Pt^IV^ succinate fragment is shown in red, the cyclic RGDFK peptide and chlorin Ce6 are displayed in blue, and the PEG linker between the two molecules in green.

Exploiting this derivativatisation route, Marchan's group attached complex **18** to a cyclic RGD peptide to target the α_v_β_3_ integrin receptor overexpressed on the surface of several cancer cells and angiogenic vasculature. The resulting bioconjugate **18‐**RGD (Figure 7) accumulated selectively and displayed antiproliferative activity in SK‐MEL‐28 melanoma cancer cells which exhibit high expression of the α_v_β_3_ integrin receptor.^56^

Another derivatization strategy consists in tethering these complexes to nanoparticulate platforms. This can improve delivery to tumors in vivo, through the enhanced permeation and retention (EPR) effect, and the nanoparticles can be further decorated with cancer‐targeting moieties and/or additional cytotoxic agents. Prominent examples of such systems include the near‐infrared photoactivatable nanoplatform developed by Xing and co‐workers, where complex **18** was attached, via the succinate moiety, to upconversion nanoparticles (UCNP), allowing the Pt^IV^ complex to be activated by near‐infrared radiation (*λ*=980 nm).^57^ Interestingly, this platform also included an apopotosis‐sensitive fluorescent peptide thus allowing imaging of the apoptotic process in living cells.

More recently, complex **13** was attached to the PDT photosensitizer chlorin e6 (Ce6) via a PEG linker.^58^ Irradiation of the resulting **13**–PEG–Ce6 system (Figure 7) not only resulted in the activation of the Pt^IV^ azido PACT agent, but also concomitant production of molecular oxygen, allowing the photosensitizer to maintain its function under hypoxic conditions. Interestingly, owing to its amphiphilic nature, **13**–PEG–Ce6 self‐assembled into micelles that, when loaded with UCNP, could be activated with near‐infrared light. This nanocomposite system was tested in vivo in xenograft tumor models, and showed effective tumor accumulation and promising anticancer activity upon NIR irradiation.

**Figure 8:**
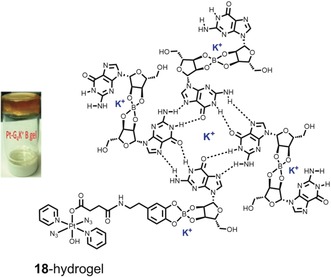
Pt(IV) hydrogel derivative **18**‐hydrogel (left), and schematic representation of its chemical structure (right). Adapted from Ref. 60.

Finally, hydrogels have been explored in our group as promising drug delivery systems because of their biocompatibility and tissue‐mimicking properties.^59^ In particular, there is potential for topical treatment of skin conditions, such as non‐melanoma skin cancer, a tissue especially accessible for PACT. Through a self‐assembly reaction, a dopamine derivative of complex **18** was incorporated into a G‐quadruplex borate hydrogel, and the resulting material (**18**‐hydrogel, Figure 8) retained the promising antiproliferative activity of its precursor complex.^60^ More extensive preclinical testing will be required to verify effective delivery of the Pt^IV^‐azido agent to cancer cells in vivo.

## 4 Challenges for Clinical Translation of Photochemotherapeutic Agents

Photoactivation at a suitable wavelength to control depth penetration, is a major consideration in the clinical translation of metal complexes for phototherapy. Ideally the complex should be activated at a wavelength whose tissue penetration matches the desired depth of the treatment. Generally, since most clinical applications require a penetration depth >1 mm, light absorbance in the red or NIR regions (including NIR‐I: 650–950 nm, and NIR‐II:1000–1350 nm) is advantageous.^1^, ^61^ On the other hand, at longer wavelengths (>900 nm) light absorption by water increases significantly (with *λ*_max_ at 960, 1440 and 1950 nm), thus limiting the range of wavelength available for PDT in the NIR‐II.^62^ Notably, some polypyridyl ruthenium complexes can achieve high red‐light PDT efficiency due to their low‐lying and long‐lived emissive intra‐ligand triplet excited states, despite their low absorption coefficients at this wavelength.^63^ Attempts to achieve longer‐wavelength photoactivation include attachment to upconversion nanoparticles^64^ and multiphoton photosensitization.^65^ However, multiphoton PDT also has limitations, posing new challenges for its clinical application. Unlike one photon PDT that can be easily carried out with low power lasers and LEDs, two photon PDT requires higher power lasers for successful photosensitization.^66^ Although the area irradiated with 2‐photon light can be highly specific, the volume of irradiated tissue is very small (≈1 μm^3^),^65^ which will necessiate new rastering techniques if wide areas are to be treated. Finally, extremely deep tissue penetration of near‐IR light activation would be undesirable for superficial lesions, such as gliomas, or non‐muscle‐invasive bladder cancer (for which TLD1433 in clinical trials with green light PDT),^67^ where reducing damage to the underlying healthy tissue is crucial.^68^ In conclusion, the ideal photosensitizer for each specific application should exhibit large molar absorption cross sections in the wavelength range that matches tumor invasion depth.

Due to their high reliance on oxygen concentration, the presence of hypoxic regions in solid tumors is another major challenge for PDT agents. Combination in the same molecule of a PDT photosensitizer and a PACT agent able to generate oxygen upon light irradiation provides a possible strategy to circumvent this issue, producing oxygen‐independent PDT photosensitizers, as recently demonstrated for **13**–PEG–Ce6.^58^ Other strategies that have been pursued include using nanocomposite systems able to catalyse H_2_O_2_ decomposition to molecular oxygen in hypoxic cancer cells.^69^ In order to reduce PDT‐induced hypoxia, photosensitizers able to inhibit cellular respiration have also been used to ensure a relatively high level of intracellular oxygen in the treated areas.^70^ Combination therapy with an hypoxia‐targeting cytotoxic agent is also able to mitigate the effect of PDT‐induced hypoxia, as recently demonstrated by Kim and co‐workers.^71^ Their novel PDT agent combines a BODIPY photosensitizer with the anti‐angiogenesis moiety acetazolamide, inhibiting carbonic anhydrase IX upregulated in hypoxic cancer cells.^71^ Finally, in recent years, the type‐I photosensitization pathway has also attracted attention for PDT in hypoxic regions. Type‐I PDT generates superoxide (^.^O_2_^−^) and hydroxyl (^.^OH) radicals as well as hydrogen peroxide (H_2_O_2_).^72^ Similar to ^1^O_2_, the highly reactive radicals ^.^OH and ^.^O_2_^−^ can react rapidly with adjacent biomolecules, disrupting normal cell functions and resulting in cancer cell death. The more stable H_2_O_2_ can diffuse across cell membranes and generate ^.^OH radicals by reaction with cellular ferrous ions (Fenton reaction) to enhance the PDT effect, especially in tumor cells with high iron demand.^73^

Tumor‐targeting efficiency is another important issue for both PDT and PACT agents, especially for systemic administration where non‐selective accumulation of these compounds can result in photosensitivity for light‐exposed organs such as skin and eyes. Attachment to tumor‐targeting vectors such as antibodies, serum proteins and cancer‐targeting peptides, can greatly increase the clinical utility of these agents by minimising side effects and reducing the amount of agent needed for a therapeutic response. Although some cancer‐targeting agents have been developed in recent years,^74^ as described in this review, their biological evaluation has mostly been limited to in vitro models and their targeting capability in vivo remains to be verified.

Finally, it is important to acknowledge that, because of the localised nature of PDT and PACT treatment, their clinical utility is intrinsically limited to localised disease. Typical indications for photoactivatable agents in oncology include premalignancies, which have not yet spread from their primary site, or small areas of recurrent or persistent cancer, where this treatment can be combined effectively with surgery and radiotherapy. A future challenge is to devise effective strategies which might allow their application to invasive diseases such as metastatic cancer and blood cancer. One way to achieve this might be to remove the need for an external light source by, for example, incorporating a chemiluminescent fragment into the photoactivatable complex, if chemiluminescence could be triggered at the tumor site. No examples of this approach have been reported so far.

## 5 Conclusions

We have focussed in this Minireview on new designs for photochemotherapeutic transition metal anticancer complexes which have emerged recently, including octahedral Ir^III^ PDT photosensitizers and Pt^IV^ PACT agents, both of which have classical inert low‐spin 5d^6^ configurations. This means that after administration, and in the dark, they are likely to reach target sites in tumors intact.

Some tris‐chelated Ir^III^ complexes, such as complex **6**, exhibit long‐lived triplet excited states and their phosphorescence not only allows them to be imaged (mapped) in cells, but also to generate destructive singlet oxygen (^1^O_2_) efficiently, even under relatively hypoxic conditions. They are photocatalysts and therefore active at low doses with good selectivity between tumor and normal cells. As well as 1‐photon activation by visible light, they can be activated by NIR radiation using 2‐photon laser methods, which increase the range of depth penetration. Surprisingly we discovered that singlet oxygen can cause specific damage to histidine residues in Hsp‐70 and aldose reductase, proteins which play important roles in cancer cells. This suggests that it should be possible to damage specific cellular target proteins by designing appropriate photosensitizers which can pre‐associate with targets. In the case of bis‐*N,C*‐chelated Ir^III^ complexes (e.g. complex **12**), we have shown that conjugation to the abundant serum protein albumin can have a dramatic effect on the transport of the photosensitizer into cancer cells, and specifically deliver the photosensitizer to the cell nucleus. This is remarkable, although relatively little is currently known about the uptake of albumin into cells. Moreover, conjugation to HSA greatly enhanced phosphorescence of the Ir^III^ complex, which again showed good selectivity between normal and human cells, and could be activated by irradiation with visible light.

Unlike PDT agents, diazido Pt^IV^ complexes are not catalysts since they decompose when photoactivated, including photoreduction to Pt^II^ and release of azidyl and hydroxyl radicals. They can also form nitrenes and generate oxygen. In contrast to clinical platinum drugs such as cisplatin and carboplatin, the photoactivated *trans* diazido complexes are more active than their *cis* analogues. These complexes induce attack on both DNA and proteins. Specific peptide damage includes oxidation of methionine and tryptophan side‐chains and interaction of released azidyl radicals with Trp generates Trp radicals. Hence their anticancer mechanism of action is quite distinct from that of cisplatin, and they have potential for treating Pt‐resistant cancers, especially surface cancers such as bladder and oesophageal. Derivatization of these complexes, via the axial hydroxido ligands allows the introduction of cancer‐targeting vectors or the conjugation to other types of delivery systems such as nanoparticles or hydrogels.

Exploration of the photochemistry and photobiology of metal complexes is still in its infancy, but it is clear that excited state complexes can affect biochemical pathways in unusual ways. However, monitoring reactions of excited state species in terms of the metal itself, its oxidation state, and the number and type of bound ligands, in time (picoseconds to hours) and in space (nm to cm), especially in living cells, is a major challenge. We can expect future progress in this field to be accelerated by further advances in the design of delivery systems for photoactive complexes (e.g. receptor targeting, multifunctional nanoparticles), light sources (e.g. multi‐photo ultrashort‐pulsed lasers, upconversion systems), light delivery methods (e.g. photonic crystal fibres),^75^ and new techniques and methods which allow target sites and mechanisms of action to be understood at the molecular and atomic level (e.g. time‐resolved luminescence imaging, nanofocussed X‐ray fluorescence). Phototherapy using metal complexes has the potential to provide new treatments for intractable diseases with fewer side effects, and hence is likely to be a highly active field for medical discoveries during the next few years.

## Conflict of interest

The authors declare no conflict of interest.

## Biographical Information

*Cinzia Imberti completed her M.Sci degree in Chemistry at the University of Padova (Italy) in 2012. She then moved to the UK to undertake a PhD at King's College London*, *where she worked on the development of new radiometal‐based radiopharmaceuticals under the supervision of Prof. Philip J. Blower. After obtaining her PhD in 2018, she joined the group of Prof. Peter J. Sadler at the University of Warwick as a Sir Henry Wellcome Postdoctoral Fellow. Her current research centres on discerning the mechanism of action and toxicity of platinum‐based anticancer agents, with particular focus on photoactivatable platinum complexes*.

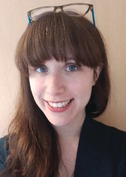

## Biographical Information

*Pingyu Zhang received her MA and DPhil degree from Sun Yat‐sen University (supervisors: Prof. Liangnian Ji and Prof. Hui Chao) in China (2010–2015). Since then she was a Newton International Fellow (2015–2017) funded by Royal Society in the Peter J. Sadler group at the University of Warwick (UK). After that, she joined Shenzhen University (SZU, China), where she is currently an associate professor and a member of “Shenzhen Peacock Talent” in Shenzhen (China). Her current research focuses on luminescent metal‐based compounds and nanoparticles in application of cancer therapy, biosensors and disease diagnosis*.

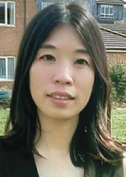

## Biographical Information

*Huaiyi Huang was born in 1988 in Guangdong, China. He received his BSc (2011) and DPhil (2016) at Sun Yat‐Sen University (China). In 2015, he became a joint PhD student in Prof. Gilles Gasser's group at the University of Zurich, Switzerland. After that, he was awarded a Newton International Fellowship to carry out research in Prof. Peter Sadler's group at the University of Warwick. He now is an associate professor at Sun Yat‐sen University. His research focuses on metals in medicine, in particular on the design and mechanism of action of therapeutic metallic complexes, including metallic photoredox catalysts, organometallic anticancer complexes and photoactivated metal complexes*.

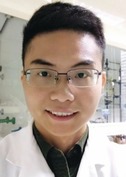

## Biographical Information

*Peter Sadler obtained his BA, MA and DPhil at the University of Oxford. Subsequently he was a MRC Research Fellow at the University of Cambridge and National Institute for Medical Research. From 1973‐96 he was Lecturer, Reader and Professor at Birkbeck College, University of London, and from 1996–2007 Crum Brown Chair of Chemistry, University of Edinburgh. Then he became Head of the Department of Chemistry at the University of Warwick, where he is now a Professor. He is a Fellow of the Royal Society of Chemistry (FRSC), Royal Society of Edinburgh (FRSE) and the Royal Society of London (FRS), and an EPSRC RISE Fellow (Recognising Inspirational Scientists and Engineers)*.